# Using social media to estimate Zika's impact on tourism: #babymoon, 2014-2017

**DOI:** 10.1371/journal.pone.0212507

**Published:** 2019-02-21

**Authors:** Mark Gallivan, Ben Oppenheim, Nita K. Madhav

**Affiliations:** Metabiota Inc., San Francisco, California, United States of America; University of Greenwich, UNITED KINGDOM

## Abstract

Zika virus infection during pregnancy can cause microcephaly and other birth defects. We hypothesized that the Latin America Zika epidemic resulted in pregnant women and their partners adopting behavioral changes to limit risk, leading them to forego travel to Zika-affected locations. We evaluated this hypothesis by studying travelers’ intent and behavior through Twitter data related to babymoon: a holiday taken by parents-to-be before their baby is born. We found the odds of mentioning representative Zika-affected locations in #babymoon tweets dropped significantly (Odds ratio: 0.29, 95% CI: 0.20–0.40) after the Zika-microcephaly association became well-known. This result was further corroborated through a content analysis of #babymoon tweets mentioning Zika-affected locations, which identified if the Twitter user was physically present in the Zika-affected locations. Conversely, we found a small but statistically insignificant increase in the odds of mentioning Zika-free locations from #babymoon tweets (Odds Ratio: 1.11, 95% CI: 0.97–1.27) after the Zika-microcephaly association became well-known.

## Introduction

The Zika virus, primarily transmitted by *Aedes aegypti* mosquitoes, was first isolated in Uganda in 1947 among non-human primates, and the first human case was identified in Nigeria in 1954 [1,2]. No locally-acquired infections were reported in the Western Hemisphere until 2015 in Brazil [3]. From 2015 to the beginning of 2018, the Pan-American Health Organization reported over 1 million cases in over 45 Western Hemisphere countries [4].

Zika virus disease typically results in asymptomatic or mild febrile illnesses, although more severe complications may occur [5]. In 2015, the Brazilian Ministry of Health (MOH) noted neurological abnormalities among babies born to pregnant Zika virus infection cases, and in January 2016, the Brazilian MOH reported a tenfold increase in the number of babies born with microcephaly (abnormal smallness of a newborn baby’s head) [6]. Over the course of the Latin America epidemic, which caused an unprecedented reported number of human infections, scientists further established that microcephaly and other teratogenic effects may arise from Zika infection during pregnancy [7].

The consequences of the Zika epidemic also went beyond its epidemiological impacts. In particular, the discovery of the association between Zika virus infection and severe, permanent birth defects reportedly altered travel and tourism patterns, causing significant economic damage to areas affected by the epidemic. A report by the United Nations Development Program found that Zika’s short-term economic impact on Latin America and the Caribbean could range between $7 - $18 billion, with significant costs driven by declines in tourism [8].

Economic assessments did not evaluate how different tourist populations reacted to the Zika epidemic. However, it would be expected that populations at highest risk of Zika-associated complications—those at risk of microcephaly, notably, pregnant women or travelers of reproductive age and/or intent—would disproportionately reduce travel to Zika-affected regions. For this reason, we explored how pregnant travelers and their partners changed their travel plans in reaction to the Zika-microcephaly association.

## Materials and methods

Statistics on international tourism are fragmented, and there is no internationally comparable body of data. Moreover, the tourism data that are available do not capture the pregnancy status of the traveler. As such, this analysis relied upon social media data drawn from Twitter to derive estimates of tourism behavior and intention for our population of interest, and search data drawn from Google to establish when Zika’s association with microcephaly became well-known among the public. This approach adds to a growing body of research using digital tools, including social media, as a means to track disease, behavior, and provide public health information [9,10]. In particular, Twitter and Google search data have been used to study many health outcomes including the forecasting of Zika incidence during the Latin America epidemic [11,12].

### Estimating when the Zika-microcephaly link became well-known

**Fig 1:**
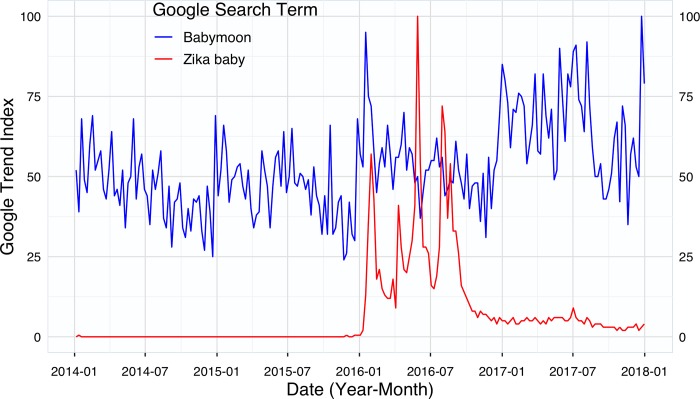
Google search trends of “babymoon” and “Zika baby”, 2014–2017.

To estimate the effect of the Zika epidemic on the behavior of travelers from non-Zika-affected areas, we first identified the time point when the Zika-microcephaly association became widely known among the public. To accomplish this task, we used Google search trend data for the term “Zika baby” from 2014 through 2017 within the United States. This term, although clearly not technical, became widely used in popular culture and Google searches (Fig 1). The term “babymoon” was also searched for in the same time period and geographic location to determine if there was any noticeable change in the term’s popularity among the general public. Google search trends provides a popularity metric of a given term among all searches performed on Google’s search engine. More specifically, the resulting output (“Google Trend Index”) is normalized against the maximum search volume for a given term over the selected time period, and allows users to evaluate the term’s relative popularity over time [13]. In July 2015, Google search queries comprised 64 percent of all searches performed online, and thus can provide a good proxy for overall online search behavior [14].

Empirical research has shown that Google trends have variable accuracy in estimating the geographic and temporal aspects of epidemiological patterns [15]. Fortunately, the term “Zika baby” was not used prior to the 2015 Latin America Zika epidemic and the term maintains a high degree of specificity that closely corresponds to an adverse outcome of Zika virus disease; therefore, the risk of misclassifying search terms is low.

The term “Zika baby” first spiked in usage in January 2016, which also coincides with several events including the first CDC-issued travel advisory targeting pregnant women, the first reported U.S. Zika-microcephaly case, the first recommendation by the European Centre for Disease Prevention and Control for pregnant women to consider postponing travel, and a Brazil MOH report indicating a substantial increase in number of Zika-associated microcephaly cases [16–18]. The World Health Organization ultimately declared Zika as a Public Health Emergency of International Concern on February 1, 2016 [19]. Sensitivity analysis of Google search terms, “Zika” and “Microcephaly”, also found an exponential rise in the terms in January 2016.

### Impact of the Zika-microcephaly link on location mentions in #babymoon tweets

Twitter is a social media platform with a global user base where users submit “tweets” (or messages) of up to 140 characters (text limit was expanded to 280 characters in November, 2017) which can be shared publicly or privately. Users can add a hashtag sign (#) in front of a term to create a meta-data index of that term for the purpose of drawing attention, organizing, and creating a conversation around that particular term.

In order to target traveling pregnant women and their partners, we utilized the hashtag babymoon. As defined by the Oxford English Dictionary, the term “babymoon” refers to “A relaxing or romantic holiday taken by parents-to-be before their baby is born” [20]. To collect Twitter data, the following search was performed through Twitter.com’s advanced search tool: tweets containing “#babymoon” written in English from January 1, 2014 through December 31, 2017 [21].

To determine how pregnant travelers and their partners changed their tourism choices, we evaluated the impact of the Zika epidemic on specific geographic locations mentioned in #babymoon tweets. We geographically coded any tweets which contained any of the location keywords (case insensitive) in the tweet itself or utilized the hashtag for the location keyword. To identify Zika-affected tourism destinations, we searched the U.S. National Travel & Tourism Office as well as epidemiological data on the origination of U.S. and Canada imported Zika cases, identified regions and localities that appeared on both lists, and selected Brazil, Mexico, and the Caribbean [22–24]. Due to the large number of potential locations, these geographic regions should be considered as representative rather than exhaustive of all potential Zika-affected tourist destinations.

Exploratory data analysis using Twitter’s advanced search tool among #babymoon tweets yielded few results with geotagged results for these Zika-affected tourist locations (and localities within these regions). Previous studies have shown approximately one percent of tweets are geotagged [25]. Furthermore, the proportion of tweets that are geotagged likely varies over time and location, which may introduce a selection bias into the sample. For these reasons, geotagged tweets were omitted from this analysis.

The Zika-free regions of Hawaii, Italy, London, Paris, and Las Vegas were also selected *a priori* because these locations have had no reported local Zika transmission and are identified as popular U.S. tourist destinations [22]. As such, we expected to observe minimal changes in the proportion of #babymoon tweets mentioning these locations.

### Analysis of Twitter data to deduce traveler behavior and intention

**Fig 2:**
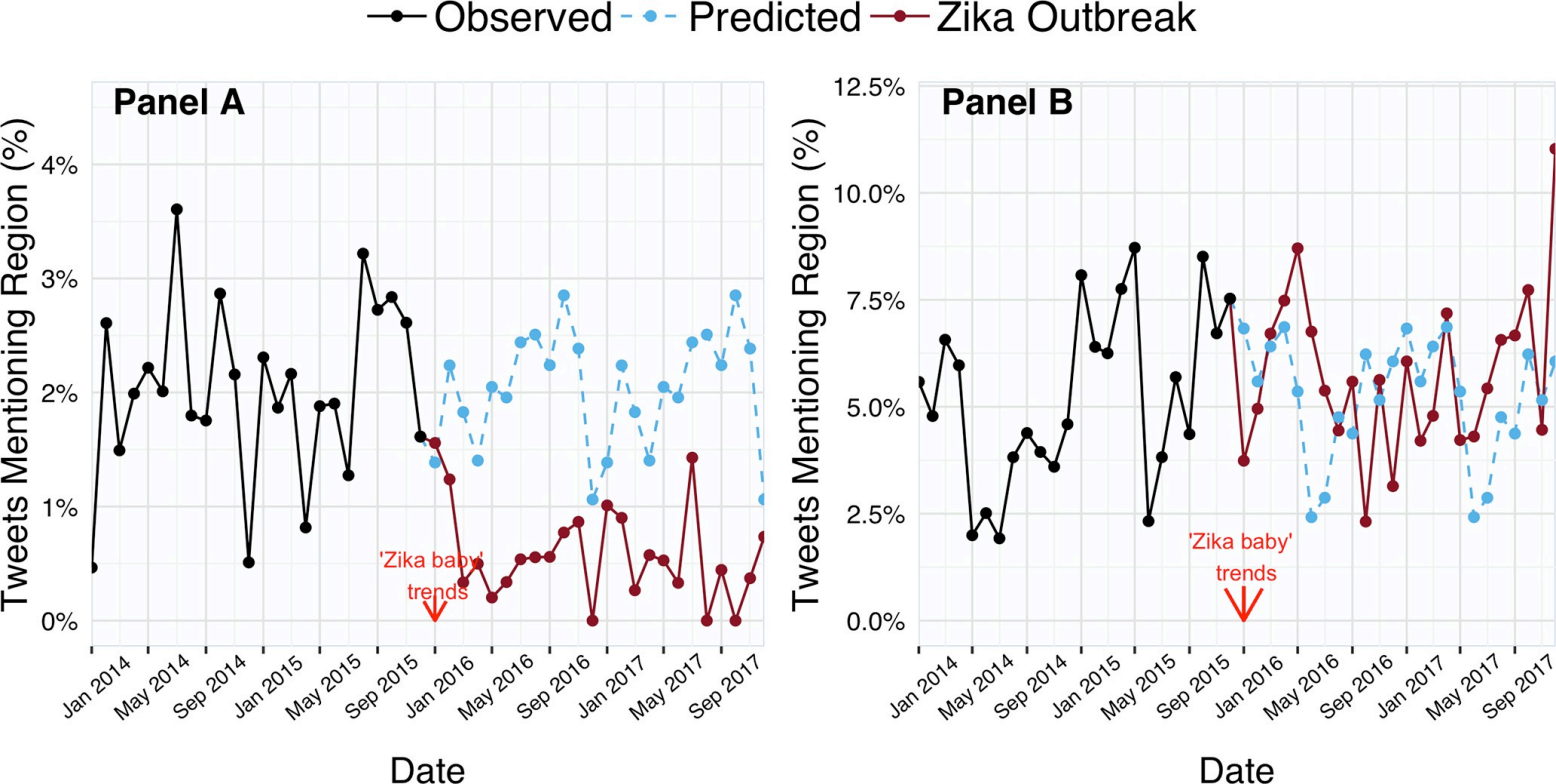
**Percent of #babymoon tweets mentioning Zika-affected (Panel A) and Zika-free regions (Panel B), 2014–2017**.

We analyzed Twitter data using several methodologies to evaluate whether the onset of public knowledge regarding Zika virus and microcephaly did indeed shift tourism intention and behavior among pregnant travelers and their partners. First, we performed chi-square tests of independence on the proportion of tweets mentioning Zika-affected locations and Zika-free locations and presented the results as odds-ratios with 95% confidence intervals. To predict what the percentage of #babymoon tweets mentioning Zika-affected and Zika-free locations would have been in the absence of the Zika-microcephaly link for 2016–2017, we calculated the mean percentage of tweets mentioning Zika-affected and Zika-free locations for each month using data from 2014–2015 (Fig 2). All statistical analyses were performed using R 3.4.2 [26]. Associations were considered statistically significant if p<0.05 (two-sided).

Second, to evaluate if the locations mentioned in tweets provide evidence the Twitter user was physically present in the Zika-affected locations or had a clear intention to travel to such locations, a random sample of 86 tweets were manually reviewed and categorized. A sample of 86 tweets was selected due to the total number (n = 43) of tweets mentioning Zika-affected regions from 2016–2017. We utilized a 1:1 ratio for both time periods (2014–2015 and 2016–2017) to ease the comparison and to maintain a tractable number of tweets to manually categorize. Given that tweets marked with #babymoon might include advertisements, public health warnings, or other types of information that could potentially increase in volume following the change in knowledge regarding Zika risk and bias our estimates, content analysis allows for screening of tweets to identify those with physical presence or travel intent and evaluate their volume over time. The tweet sample was stratified by post date of the tweet, with an equal number selected from the timeframes before and after the Zika-microcephaly link became well-known. The coder was blinded to the post date of the tweet and categorized tweets into four categories using the coding guidelines given in Table 1.

**Table 1:** Content analysis categorization guidelines.

Category 1: Physical presence	Category 2: Advertisement	Category 3: Warning of Zika-microcephaly link	Category 4: Other
- Tweet described events occurring in a particular locality - Tweet described presence at a specific locality - Tweet described future plans to visit a specific locality	- Tweet described, referred to, or linked to travel or hospitality property	- Tweet described Zika symptoms, health risks, disease transmission - Tweet provided information on Zika risk mitigation	- Tweet only contained relevant keyword, with no other related information - Tweet was a seemingly random "bag of words": garbled text with no syntactical structure or substantive content

## Results

### Estimating when the Zika-microcephaly link became well-known

Google search data show that the term “Zika baby” was not in public use prior to late 2015 and rose precipitously in January 2016 (Fig 1 and S1 Dataset). Since late 2016, the term has maintained a small but discernible share of search activity. Conversely, according to Google search trend data in the United States, the term “babymoon” has maintained popularity since 2014 without a clear trend in the term’s usage over time.

### Impact of the Zika-microcephaly link on location mentions in #babymoon tweets

We extracted a total of 15,818 #babymoon tweets posted between January 1, 2014 and December 31, 2017 (Table 2). Of the total 15,818 tweets, 8,564 were posted before the Zika-microcephaly link became well-known and 7,254 were posted after the Zika-microcephaly link became well-known. A significant drop in the percentage of #babymoon tweets mentioning Mexico, Brazil, and the Caribbean occurred after the Zika-microcephaly association became well-known (Fig 2, Panel A). The monthly mean interpolation model predicted that during August 2016, approximately 2.5% of #babymoon tweets would mention Mexico, Brazil, or the Caribbean; however, the observed percentage was less than 0.6%. In addition, the odds of mentioning Zika-affected locations in #babymoon tweets were 0.29 (95% CI: 0.20–0.40) times as likely after the Zika-microcephaly association became well-known (Table 2).

**Table 2:** Location mentions in #babymoon tweets, 2014–2017.

Location Mentions	2014–2015: before Zika-microcephaly link (n = 8564)	2016–2017: after Zika-microcephaly link (n = 7254)	Odds Ratio (95% Confidence Interval)
**Zika-affected locations (total)**^**a**^	**175**	**43**	**0.29 (0.20–0.40)**
Brazil	2	5	
Caribbean	52	17	
Mexico	136	32	
**Zika-affected locations not mentioned**	**8389**	**7211**	
**Zika-free locations (total)**^**a**^	**442**	**413**	**1.11 (0.97–1.27)**
Hawaii	249	203	
Italy	67	77	
London	49	62	
Paris	64	72	
Vegas	74	75	
**Zika-free locations not mentioned**	**8122**	**6841**	

^**a**^ **Sum of individual locations may not equal Zika-affected or Zika-free location total if a tweet mentions multiple locations**

Conversely, the odds of mentioning Zika-free locations in #babymoon tweets were 1.11 (95% CI: 0.97–1.27) times more likely after the Zika-microcephaly association became well-known (Fig 2, Panel B).

### Content analysis of tweets to validate traveler behavior and intention

During 2014–2015, 23 out of 43 (53.4%) tweets mentioning Zika-affected locations had evidence the user was physically present at the location or expressed intentions to visit the location (Table 3). In contrast, between 2016–2017, 13 out of 43 (30.2%) tweets had evidence the user was physically present at the location or had intentions to visit the location, which was statistically significant compared to the pre-Zika proportion (p = 0.049). Approximately 12% of the #babymoon tweets mentioning Zika-affected locations during 2016–2017 contained messages warning of the Zika-microcephaly link. No tweets indicated the Twitter user was a resident of Brazil, Mexico, or the Caribbean. Additionally, a manual review of all tweets mentioning Zika-affected or Zika-free locations containing the word “not” found no instances that the user indicated they were *not traveling* to that locale.

**Table 3:** Content analysis of sampled #babymoon tweets mentioning Zika-affected locations, 2014–2017.

	2014–2015: before Zika-microcephaly link (n = 43)	2016–2017: after Zika-microcephaly link (n = 43)
Physical presence	23 (53.4%)	13 (30.2%)
Advertisement	12 (28.0%)	18 (41.9%)
Warning of Zika-microcephaly link	0 (0.0%)	5 (11.6%)
Other	8 (18.6%)	7 (16.3%)

## Discussion

We found a significant reduction in the proportion of tweets mentioning selected representative Zika-affected locations after the Zika-microcephaly link became widely known among the public. Conversely, a small but statistically insignificant increase was observed in the proportion of tweets mentioning selected Zika-free locations, suggesting travelers may have stayed home or traveled to another location. Through a content analysis of babymoon-related tweets, we found a statistically significant decline in social media activity that suggested physical presence in a particular Zika-affected area after the Zika-microcephaly link became well-known. Together, these two analyses provide evidence that pregnant travelers and their partners reduced travel to Zika-affected locations in response to the Zika-microcephaly link. This response can likely be interpreted as a precautionary reaction to mitigate the risk of birth defects.

Relatedly, our content analysis of #babymoon tweets mentioning Zika-affected locations found a modest, but statistically insignificant, increase in the number of tweets which contained advertising for Zika-affected areas after the Zika-microcephaly association became widely known. The increase may reflect a reaction by economically-impacted tourism businesses, attempting to use advertising specifically targeted at potential pregnant travelers and their partners to mitigate declines in tourism arrivals and spending.

Previous studies on Zika-related knowledge and attitudes among reproductive-aged women have showed mixed results in terms of pregnant traveler knowledge and behavior. A survey conducted in Miami found that most reproductive-aged women knew of the Zika-microcephaly association [27]. However, another survey conducted around the same time period found that 43 out of 99 New York City women who traveled to areas with active Zika virus transmission while pregnant were unaware of the potential risk inherent to their travel [28]. While these two surveys demonstrate substantial geographic disparity in public knowledge about Zika virus among pregnant women within the United States, the macro-level picture yielded by our analysis of social media patterns suggests that knowledge of Zika risk—and behavioral adaptations to that risk—may be widespread. As social media remains an important source for the dissemination of public health information, educational outreach on social media may benefit from utilizing relevant hashtags, like #babymoon, to reach a targeted audience with elevated risk factors [9].

### Limitations

This analysis contains a few limitations. We assume that mentions of geographical locations are a proxy measure of physical presence at that location or intention to travel there. Future studies should consider using the full Twitter archive in conjunction with geotagged tweets and explore the effect of the Zika epidemic on different geographic localities. Our investigation into this limitation through a blinded manual content analysis of sample tweets found that approximately half of the tweets mentioning Zika-affected locations indicated the Twitter user was either physically present at the location or intended to visit those locations. This limitation would have minimal impact on the association of Zika-microcephaly and mentions of locations in #babymoon tweets because the potential bias would be present throughout the study period. Additionally, the content analysis of Zika-affected location #babymoon tweets found a differential misclassification that would bias the odds ratio away from the null effect.

Another limitation comes from the assumption that the Twitter user resided outside the selected Zika-affected and Zika-free locations. For the Zika-affected locations, this is mitigated because the query only contains tweets in English, which is not the predominant language of Brazil, Mexico, and the Caribbean. Furthermore, there was no evidence that the user resided in the Zika-affected locations in the content analysis, and the term babymoon contains much higher variance in Google search trends for Brazil and Mexico, suggesting the term is less popular in those locations compared to the United States [29].

## Conclusions

In our study, we utilized a Twitter hashtag, #babymoon, associated with traveling pregnant women and their partners, to evaluate tourism behavior change for a population that would understandably be very responsive to the Zika-microcephaly link. Through social media activity and content analysis of #babymoon tweets, we find evidence suggestive of a large travel decline to Zika-affected locations. This study highlights an application of social media data to help track epidemiologically-relevant behavior across time, and understand when health-related knowledge enters the public’s consciousness. Such knowledge can help us to better understand societal reaction to public health risks, and to support effective risk communication to improve public health.

## Supporting information

S1 Dataset Data used in Fig 1. Variables include Year_Month, Babymoon (mean Google Trend Index score for “babymoon”), Zika_baby (mean Google Trend Index score for “zika baby”), Zika (mean Google Trend Index score for “Zika”), and Microcephaly (mean Google Trend Index score for “microcephaly”).
(CSV)
